# The informed road map to prevention of Alzheimer Disease: A call to arms

**DOI:** 10.1186/s13024-021-00467-y

**Published:** 2021-07-21

**Authors:** Eric McDade, Jorge J. Llibre-Guerra, David M. Holtzman, John C. Morris, Randall J. Bateman

**Affiliations:** 1 Department of Neurology, Washington University in St Louis, 660 S. Euclid Avenue, Campus Box, St Louis, MO 8111 USA; 2 Knight Alzheimer’s Disease Research Center, Washington University School of Medicine, St. Louis, MO 63110 USA; 3 Dominantly Inherited Alzheimer’s Network Trials Unit, St. Louis, MO 63110 USA

**Keywords:** Alzheimer disease, Clinical trials, Primary and secondary prevention

## Abstract

Alzheimer disease (AD) prevention trials hold the promise to delay or prevent cognitive decline and dementia onset by intervening before significant neuronal damage occurs. In recent years, the first AD prevention trials have launched and are yielding important findings on the biology of targeting asymptomatic AD pathology. However, there are limitations that impact the design of these prevention trials, including the translation of animal models that recapitulate key stages and multiple pathological aspects of the human disease, missing target validation in asymptomatic disease, uncertain causality of the association of pathophysiologic changes with cognitive and clinical symptoms, and limited biomarker validation for novel targets. The field is accelerating advancements in key areas including the development of highly specific and quantitative biomarker measures for AD pathology, increasing our understanding of the course and relationship of amyloid and tau pathology in asymptomatic through symptomatic stages, and the development of powerful interventions that can slow or reverse AD amyloid pathology. We review the current status of prevention trials and propose key areas of needed research as a call to basic and translational scientists to accelerate AD prevention. Specifically, we review (1) sporadic and dominantly inherited primary and secondary AD prevention trials, (2) proposed targets, mechanisms, and drugs including the amyloid, tau, and inflammatory pathways and combination treatments, (3) the need for more appropriate prevention animal models and experiments, and (4) biomarkers and outcome measures needed to design human asymptomatic prevention trials. We conclude with actions needed to effectively move prevention targets and trials forward.

## Background

In the absence of highly effective disease-modifying treatments and against a backdrop of an aging population, the number of adults with dementia worldwide is projected to more than triple by 2050 [1–3]. A prevention treatment delaying the onset of Alzheimer disease (AD) dementia by five years would result in a 41% lower prevalence and a reduction in personal and societal costs of about 40% [4, 5].

To date, trials in persons with symptomatic AD (a term that encompasses mild cognitive impairment due to AD and AD dementia) [6] largely have targeted amyloid-beta (Aβ), the earliest contributor to AD pathophysiology [7–9]. Many of these trials had major limitations, including little impact on biology (*too little*) or treating symptomatic AD after neurodegeneration and tau pathology is advanced (*too late*). Some classes, such as β-site amyloid precursor protein cleaving enzyme (BACE) inhibitors and Aβ antibodies, substantially engaged their targets, but failed to demonstrate a clear clinical benefit in phase 3 trials of symptomatic AD [10–16].

Identifying and demonstrating clear disease-modifying treatments for AD has continued to be elusive. Until drug classes demonstrate a consistent substantial change in the clinical disease course, potential targets are both numerous and uncertain and include Aβ (plaques, surrounding protofibrils/oligomers, monomers, and Aβ modifications such as pyroglutamate, truncations, and amino acid substitutions), tau (tangles, oligomers, seeding, aggregation, phosphorylation, acetylation), inflammation (microglia, activated astrocytes, complement), neurodegeneration (protein homeostasis, vascular transport mechanisms, cytokines), apolipoprotein E (which impacts Aβ, tau, inflammation, and neurodegeneration), and the neurovascular unit (capillary/neurons/astrocytes/pericytes). Ongoing AD trials seek to engage these myriad targets but are limited in number, speed, and scope, resulting in too few shots on goal. Many newly developed drugs do not proceed to clinical trials due to the uncertainty of success combined with the untenable cost, duration, and size of trials, which sequester pharma resources [17].

Another major issue with current AD trials may be related to the selection of participants *too late* in the disease course or incorrect target selection according to disease stage. Further, even if current symptomatic trials are successful with the targeted goals of 25–40% slowing of disease progression, millions of patients and families will still have to endure slow progressive loss of cognitive abilities and of daily function, resulting in increasing disability and dependence. Therefore, it would be optimal to intervene before substantial cognitive impairment has occurred. Advancements are needed to improve the chances of clinical trial success, including improved target and drug mechanism validation, identification of those at risk of AD, rapid screening and enrollment into prevention trials, and clear proof of concept studies that demonstrate significant biological engagement [18, 19].

**Fig. 1:**
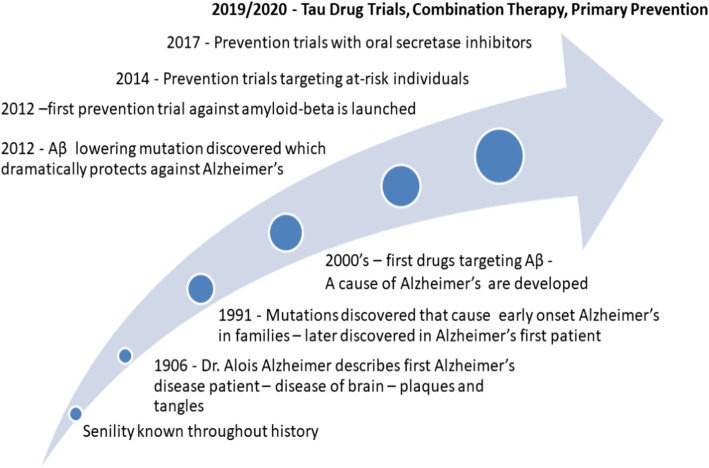
A brief history of AD prevention trial development

**Fig. 2:**
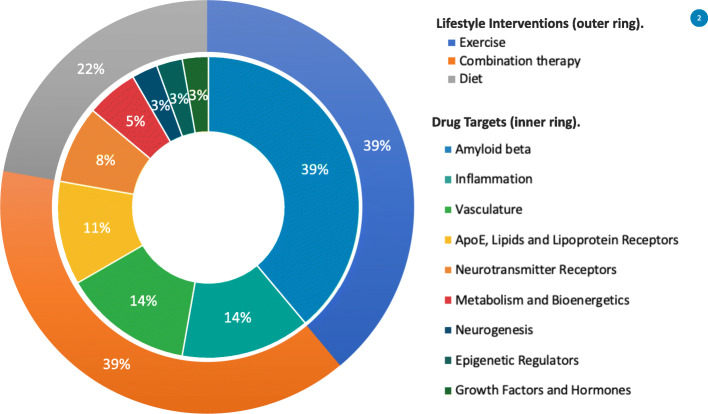
Alzheimer’s disease prevention trials and targets: Prevention trials and target agents for treatment of Alzheimer’s disease in 2020 (from ClinicalTrials.gov as of August 24, 2020). The inner ring shows prevention trials agents; the outer ring presents lifestyle prevention trials. AD therapeutics interventions were classified according with the terminology of the Common Alzheimer’s and Related Dementias Research Ontology (CADRO). Lifestyle interventions (*N* = 18), Drug Targets (*N* = 37)

The AD field is rapidly advancing our ability to implement these improvements with recent discoveries in identifying pre-symptomatic stages of AD, quantitative biomarkers to measure the magnitude of disease modification, and simple and fast screening blood based biomarkers to identify at risk individuals [20–22]. The recent development of biomarkers to track AD pathology [23–25] has led to a better understanding of the AD spectrum of disease stages, having significant implications for early treatment and prevention trial enrollment and for back-translation to preclinical studies. As a result, the search for therapeutic agents has moved earlier in the disease continuum into the asymptomatic stage of AD, which is defined by the presence of AD pathology without clinically apparent symptoms, and the field has launched the first generation of prevention trials (Fig. 1). We define primary prevention as an intervention that is implemented before evidence of disease or injury (i.e., before amyloid or tau pathology or neurodegeneration). Secondary prevention is treating pathologic disease in asymptomatic individuals with subclinical forms of the disease (e.g. cognitively unimpaired but AD biomarker-positive, identifying those with increased risk for future symptomatic AD). Several primary and secondary AD prevention trials currently are in the planning stages, have begun enrolling subjects, or have just concluded (Fig. 2). Although operational implementation of AD prevention trials has been feasible, multiple challenges need to be addressed to optimize trial design. These include validation of targets for primary or secondary prevention stages of the disease, improved understanding of biomarker progression leading to clinical symptoms, accurate translation of animal and *in vitro* models to human studies, and development of models for AD-related inflammation. Here, we review the current field of prevention trials by therapeutic target and propose key areas of research needed as a call to basic and translational scientists to improve the design and interpretation of AD prevention trials.

## Review of amyloid prevention approaches

The amyloid hypothesis proposes that amyloid plaques formed by aggregates of the Aβ peptide generated by proteolytic cleavage of amyloid precursor protein (APP) are central to AD pathology [26]. Increased levels or ratios of Aβ42 induce Aβ amyloid fibril formation [27] and, in late onset AD, impaired clearance of Aβ leads to the age-associated risk of amyloidosis and AD [28]. The accumulated Aβ amyloid fibrils develop into senile plaques, causing local, plaque-associated neurotoxicity and later facilitation of the spread of tau pathology [29]. The oligomeric and protofibrillar species are hypothesized to be the most toxic forms [30] and may facilitate tau hyperphosphorylation, disruption of proteasome and mitochondrial function, dysregulation of calcium homeostasis, synaptic failure [31], and glial cell activation [31, 32]; leading to neuronal cell death, neurodegeneration, and cognitive impairment. However, it is unlikely that Aβ accumulation directly leads to marked synaptic loss and neuronal cell death as substantial accumulation of Aβ occurs in the human brain and in animal models of Aβ accumulation without significant synaptic loss and neuronal death. Aβ aggregates induce downstream changes such as tau accumulation and inflammation that are more proximate to synaptic failure and cell death. Aβ accumulation appears to be the first pathogenic event leading to subsequent downstream pathological changes and remains an important target of AD prevention trials.

The amyloid hypothesis has been tested in multiple therapeutic strategies aimed at targeting Aβ in the brain. The approaches include Aβ active or passive immunization [33–37], and γ- and β-secretase inhibitors or modulators [10, 38, 39]. Despite the scientific strength of the amyloid hypothesis, to date clinical trials targeting Aβ have failed to clearly demonstrate clinical benefit [14, 36] with the consequence that the amyloid hypothesis has been questioned [7, 40, 41]. However, there are several reasons that may account for these trial failures including treating *too late* in the disease course, inadequate dosing or target engagement, and incorrect specific Aβ target (e.g. monomer, oligomer, or plaque) [40, 42].

Because amyloid deposition is one of the first events beginning 20 years or more before AD dementia, administering Aβ therapies at symptomatic or advanced stages of ongoing neurodegeneration may provide little clinical benefit. Aβ therapeutic agents are ideal candidates for primary or secondary prevention strategies before the development of substantial tau tangles, inflammation, and neurodegeneration as they may prevent the onset of dementia. This concept is supported by recent results from the Dominantly Inherited Alzheimer Network Trials Units (DIAN-TU), where significant reduction of brain amyloid deposition by gantenerumab, a monoclonal antibody targeting aggregated Aβ, had strong effects in downstream biomarkers. Compared with placebo, participants treated with gantenerumab showed significant reductions in CSF total tau and p-tau181 and slowed increases in CSF NfL. In support of the concept of a need for earlier intervention of Aβ therapeutics, the effect of gantenerumab on plaque reduction and downstream biomarkers of tau pathology and neuronal injury appeared to be larger in the asymptomatic group vs. the symptomatic group—though the small number of participants limited the statistical power of these findings. Although no cognitive or clinical benefit was observed, these findings suggest that removal of amyloid plaques may be a viable strategy in preventing or slowing the biological progression of AD if introduced at an optimal time [43].

Trials aimed to prevent Aβ aggregation will require many years to obtain biomarker readouts and even longer to determine if prevention of Aβ aggregation delays or stops symptomatic AD phases. Primary prevention trials will require participant enrollment for long periods and academic, private, and government partnerships will be key elements for the development and successful completion of such trials. Several efforts are underway for primary prevention trials, including DIAN-TU, which will use anti-Aβ agents in the world’s first primary prevention trial in AD. Because of the uncertain outcomes of AD prevention studies, preclinical studies that can minimize risk by defining the optimal time to introduce amyloid specific therapies or the expected effects on tau and inflammation by preventing or removing amyloid plaques are high priority goals.

Table 1 summarizes ongoing and planned Aβ prevention trial therapies that are currently being tested in symptomatic stages but that also have a strong rationale for application in prevention studies. Given the large number of Aβ specific clinical trials that have been conducted without clear success to date, below we summarize priorities for animal model studies to increase the probability of success in AD prevention studies [46].

**Table 1:** Current Aβ therapeutics with rationale for AD prevention clinical trials

Class	Compound	Current status	Disease Stage	Trial #/Names	Sample size	Trial Outcome measures	Evidence from clinical studies
Immuno-therapy	ABvac 40 (active vaccine)^a^	Phase 2	Mild AD	NCT03461276	120^b^	Safety and immune response	>90% immune response [44]
	ACI-24 (active vaccine)	Phase 2	Asymptomatic Down Syndrome	NCT04373616 ACI-24-0701	72^b^	Safety and MRI	Initial formulation with suboptimal immune response
	BAN2410	Phase 2/3	Asymptomatic with low or higher amyloid plaque load	NCT01767311 AHEAD (A345)	856	Safety and Cognitive change	Active, results not yet available
	CAD106	Phase 3	Asymptomatic sporadic AD	NCT02565511	480	Cognitive change	Terminated, results not yet available
	Crenezumab	Phase 2	Asymptomatic DIAD	NCT01998841	252	Cognitive change	Low to High dose being tested in Colombian kindred (PSEN-1_E280A)
	Gantenerumab	Phase 2/3	Asymptomatic DIAD; Mild AD	NCT01760005 NCT03443973	73 982	Biomarker, Cognitive, and Clinical change	Tau and neurodegeneration biomarker improvements with lowered CSF tau, p-tau181, and NfL. No clinical benefit in DIAD at low dose. High dose continued testing in DIAN-TU OLE prevention trial
	Solanezumab	Phase 3	Asymptomatic DIAD completed Asymptomatic sporadic AD	NCT01760005 NCT02008357 A4	71 1150^b^	Biomarker, Cognitive, and Clinical change	No tau or neurodegeneration biomarker improvements or clinical benefit in DIAD on low dose; asymptomatic sporadic AD prevention trial (A4) ongoing with higher dose
	UB-311 (active vaccine)^a^	Phase 2	Mild AD	NCT02551809	43	Safety and immune response	>90% immune response with a good safety profile.
Small Molecule^c^	PQ912 (Inhibitor of glutaminyl cyclase)	Phase 2	Early AD	NCT03919162	414^b^	Safety, PK and clinical outcomes	Good safety profile; trends for cognitive benefit [45].
	BACEi JNJ-54861911 MK-8931 (verubecestat) E2609 (Elenbecestat) CNP520 LY3314814 Lanabecestat	Phase 3	Asymptomatic/Early AD	NCT02569398 NCT01953601 NCT03036280 NCT03131453 NCT02245737	557 1454 2212 1145 2218	Negative effects in cognition, decrease Aβ	Clinical studies halted due to safety concerns with rapid mild negative effects in cognition which may be reversable.

The table represents the recent approaches to Aβ therapeutics highlighting new mechanisms to be tested on prevention trials

^a^Potential agents for primary prevention

^b^Potential agents for primary/secondary prevention. Proposed potential as a prevention therapy is based on how likely the known mechanism of action aligns with the disease stage of Aβ-pathology (prevent Aβ aggregation or Aβ plaques removal), the known side effect profile (long term treatments likely required) and the available data on clinical efficacy

^c^Estimated enrollment

### Animal models re-create some, but not all aspects of AD in humans

Nearly 200 mouse models that have various aspects of AD pathology (https://www.alzforum.org/research-models/search) have been generated to explore disease pathophysiology and identify various therapeutic strategies. Animal models constitute one of the most important research tools in basic research for AD and have resulted in key findings for AD. Current models either develop Aβ plaques or neurofibrillary tangles (NFTs), or in some cases a combination of both pathologies, but these transgenic mice do not exhibit the full spectrum of AD pathophysiology [47, 48]. The amyloid pathology is different in AD models compared to the human AD pathology, with differences in ultrastructure, amount and kinds of amyloid and in the binding to amyloid PET tracers. Moreover, these models do not replicate the full range of co-pathologies often present in sporadic AD, including vascular disease, Lewy body disease, TDP-43, or hippocampal sclerosis. Therefore, current models do not recapitulate the complexity of late-onset AD. In addition, sporadic AD arises from the interaction between genetic and environmental factors, but current models are based on genetic mutations and do not replicate key lifestyle risk factors linked to symptomatic onset and disease progression. These differences between AD models and human AD may partly explain how therapeutic strategies that clearly work in mouse models do not replicate the pathological findings in the model or translate into positive results in human AD clinical trials. Finally, many of the interventional studies with AD models implement treatment at a much earlier stage, usually before substantial pathology is established, while human clinical trials in symptomatic AD implement intervention only after the amyloid-induced pathological cascade has been established.

### Timing of Aβ intervention

**Fig. 3:**
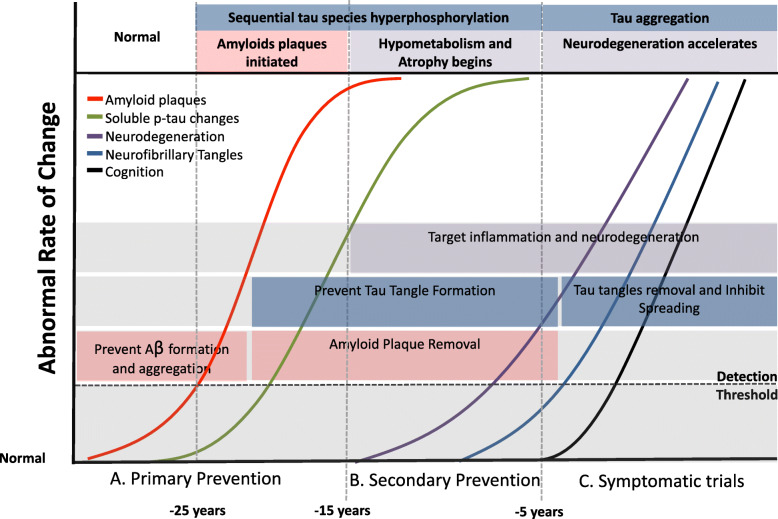
Timing of AD prevention trials related to core pathology and symptom onset

A major issue with the failure of some Aβ interventions may be related to enrolling participants too late into the disease course (i.e., when symptomatic AD is manifest, even at its earliest stages), when neurodegeneration and synapse loss already has occurred and Aβ pathology has been fully established over a after two-decade period of growth. At these symptomatic stages, Aβ plaque removal may have little effect on cognition. Ideally, anti-Aβ agents should be introduced early enough to avoid tau aggregation and the alteration of the CNS inflammatory response as, once initiated, these processes may be autonomous from amyloid. The concept of initiating Aβ interventions in primary and secondary prevention trials before later stages of AD is shown in Fig. 3.

In order to inform the design of these prevention studies, AD mouse models are needed that recapitulate the human disease of sequential progressive steps of amyloid, soluble tau, hypometabolism, atrophy, aggregated tau and tangles culminating in neurodegeneration, cognitive and clinical impairment resulting in death. Models which can better recapitulate the sequential stages of human AD should then be used to test interventions *before, during, and after* pathological target development to inform about the stage of human disease to design clinical studies. Few interventions targeting Aβ in animal models were tested to determine if they remove existing fully established amyloid plaques as opposed to determining if they prevent further increases in plaques. A recent study highlighted this potential utility by demonstrating that APP secretase inhibitors were primarily effective in preventing rather than significantly reversing Aβ plaque pathology [49]. Had there been similar studies for other secretase inhibitors (γ and β), clinical trials could have been designed toward earlier prevention stages and possibly utilized lower dosing, which is a key factor, as the secretase inhibitors have safety issues that may be dose dependent.

### Summary

Based on the multitude of clinical trials that have been conducted with Aβ targeted approaches, Aβ therapies do not have a large clinical effect in symptomatic AD but may delay or even prevent symptomatic AD, if introduced sufficiently early in the asymptomatic disease process. Recent findings with immunotherapies indicate that targeting specific types of fibrillar Aβ [50, 51] can have a large biological effect in asymptomatic prevention trials [52]. However, much remains to be understood with these interventions, particularly whether the clinical benefit is mediated from a direct amyloid reduction, indirect reduction of soluble or aggregated forms of tau, or an interaction with the CNS immune system and slowing neurodegeneration. Basic and translational sciences should accelerate development of appropriate amyloid prevention models, identify amyloid and downstream prevention biomarkers, and test amyloid interventions under conditions that enables translation to prevention trials.

## Review of Tau prevention approaches

Hyperphosphorylated, intracellular insoluble tau in the form of NFTs is pathognomonic of AD [53, 54]. Tau pathology in the brain correlates much more highly with clinical status and dementia than do amyloid plaques, atrophy, or perturbations of glucose metabolism [55, 56]. Tau aggregation correlates with onset, progression of cognitive symptoms, and focal neurologic symptoms (e.g., memory, visuospatial function and language) [57]. CSF total-tau (i.e. all soluble tau regardless of phosphorylation) and phosphorylated-tau (i.e. p-tau with specific phosphorylation sites) isoforms predict clinical onset and tau in blood mirrors these results [58]. A molecular structural description of tau pathology demonstrates identical structures in Dominantly Inherited AD (DIAD) and sporadic AD (sAD), which differs from non-AD tauopathies [59]. Normal forms of tau function as a regulator of axonal remodeling are actively regulated and produced, and extracellular tau has increased production correlating with amount of amyloid plaques [60]. Importantly, as it relates to prevention trials, tau soluble changes begin near amyloid plaque pathology, 20 years before symptom onset, while the aggregated tau pathology measured by tau PET appears at symptom onset (see Fig. 3) [21, 61–63]. This provides a window of opportunity to intervene in tau before symptom onset and the growth of tau tangle aggregation.

Tau targets present challenges due to the complexity of the tau target and pathological changes with a large number of post-translational modifications, including phosphorylation, and dozens of fragments and alternative forms [64]. Unfortunately, the leading mouse models that develop tau pathology (e.g., P301S, P301L) used in preclinical testing of anti-tau therapies have frontotemporal lobar degeneration (FTLD) tauopathies which are structurally different from AD tauopathy, limiting their predictive power for AD clinical trials [65, 66]. Further, we know much less about the longitudinal changes of tau pathology due to limited kinds of biomarkers and lack of understanding of the relationship between the soluble tau measures in CSF and blood and the aggregated tau measured by tau PET. For example, the tau in CSF is N-terminal to mid-domain, while the tau present in tau aggregates and NFTs are comprised of the C-terminal region, so they are distinctly different parts of the protein and pathophysiology.

Our understanding of sequential changes in tau biomarkers identifying tau stages in DIAD and sAD has improved over the past few years, enabling the ability to detect drug effects on biomarker outcomes [67, 68], including at the prevention stage. A near-term need is to improve target validation of tau in the asymptomatic versus symptomatic stages and to understand the relationship between soluble tau and tau aggregation. One possibility is that aggregation of amyloid causes a physiologic response of increased tau production and soluble secretion of hyperphosphorylated forms that increases the risk of tau intracellular aggregation some two decades later that is manifest at the time of progression to symptomatic AD. The diverse mechanisms by which aggregated tau may directly or indirectly be related to neuronal toxicity require additional research in order to improve the predictability of therapies targeting aggregated forms of tau.

### Tau therapies: current state

In addition to the strong rationale for targeting tau to treat AD (and other neurodegenerative tauopathies), the lack of success of Aβ-specific therapies has intensified the search for tau therapies. Recent comprehensive reviews of tau therapies provide in-depth assessments [69]. Table 2 summarizes current approaches to tau interventions with a focus on prevention mechanisms to prevent or remove hyperphosphorylated or aggregated tau. Lowering soluble forms of tau is not synonymous with removing aggregated tau, and provides a twenty year window of opportunity to prevent the formation of intracellular NFTs as the key pathological form of tau in AD [76].

**Table 2:** Tau therapeutics in clinical trials

Class	Compound	Current status	Disease Stage	Trial #	Sample Size	Preclinical models used	Trial Outcome measures	Opportunity for prevention^a^	Evidence from clinical studies
Immuno-therapies (active vaccine and passive antibodies)	**AADvac1**—active vaccine against truncated N-terminal tau	Phase 2 (mild AD)	Mild AD	NCT02579252	208	Human truncated tau cDNA-rat	Safety;	Yes	Slowing of NfL rise; possible slowing of MRI atrophy and CSF p-tau
	**ABBV-8E12**—passive; N-terminal of aggregated tau	Phase 2	Mild AD PSP	NCT02880956	453	P301S	AD-ongoing PSP-clinical progression	Possible	Phase 2 study in PSP demonstrated no clinical/imaging benefit at interim
	**BIIB076**—passive; N-terminal mid-domain tau	Phase 1	AD	NCT03056729	46	P301L	Safety	Possible	Phase 1 completed-results pending
	**Gosuranemab**—passive; N-terminal extracellular tau	Phase 2	AD PSP	NCT03352557	654	ADAD iPSC; P301L	Safety;	Yes	Phase 2 study in PSP demonstrated no clinical
	**JNJ-63733657**—passive; mid-domain (tau seeds)	Phase 1	Mild AD	NCT03375697	72	P301L	Safety	possible	Safety study in mild AD completed; results pending
	**Semorinemab**—passive; n-terminal (monomeric/oligomeric)	Phase 2	Mild AD	NCT03828747 NCT03289143	260^b^ 457	P301L	Clinical/Cognition	possible	Phase 2 study reported no effect on cognitive decline
Microtubule stabilizer	**epothilone D**—microtubule stabilizer (taxane derivative) [70]	Discontinued	Mild AD	NCT01492374	40	P301L P301S	Safety/CSF biomarkers	Unknown	Phase 1b study completed but no results provided
	**TPI287**-tubulin-binding and microtubule-stabilizing (taxane derivative)	Phase 1	AD PSP CBS	NCT01966666	29	NA	Saftey/CSF PK	Unknown	Lack of clear clinical benefit for all tauopathies tested; increased anaphylactoid reaction in AD [71]
Tau Aggregation inhibitor	**Anle138b**—general inhibitor of protein aggregation [72]	Phase 1	Healthy adults	NCT04208152	68	P301S [73] hTau [72]	Safety	Possible	Good safety in phase 1 healthy adults
	**LMTM**-second-generation tau protein aggregation inhibitor (Aminothienopyridazines) [74]	Phase 3	AD FTD	NCT03446001	588^†^	Recombinant Tau/cellular models	Clinical/cognitive decline	Possible	Multiple phase 2/3 studies with multiple formulations/doses have failed to identify a clear clinical benefit
Phosphorylation/protein kinase inhibitors/dephosphorylation enhancers	**Lithium**—multiple but some glycogen synthase kinase 3 (GSK3-β) inhibition	Phase 2	Mild AD	NCT01055392	80^b^	NA	Clinical/cognition	Unknown	Possible stabilization of cognition in mild AD [75]
	**Tideglusib**-inhibitor of glycogen synthase kinase 3 (GSK3-β)	Phase 2	AD PSP	NCT01350362 NCT00948259	306	Double transgenic APP/Tau	Safety/Clinical improvement	Unknown	No clinical benefit
Non-specific	**Davunetide**—activity-dependent neurotrophic protein; decreased tau phosphorylation	Discontinued	AD PSP	NCT00422981	144	ADNP transgenic mice	Safety/clinical	No	No clear cognitive benefit
MAPT lowering (Antisense Oligonucleotide)	**BIIB080**	Phase 2	AD	NCT03186989	46	P301S	Safety/Clinical	Yes	TBD

The table represents the recent approaches to tau therapeutics highlighting the focus on mechanisms based primarily from preclinical models

*AD* Alzheimer disease, *CBS c*orticobasal syndrome, *PSP* progressive supranuclear palsy

^a^Proposed potential as a prevention therapy is based on how likely the known mechanism of action aligns with the earliest stages of tau-pathology (soluble extracellular tau, reversibility of initial aggregated pathology), the known side effect profile (long term treatments likely required) and the available data on clinical efficacy

^b^Estimated enrollment

With recent advances in the understanding of tau, multiple novel tau therapeutics have advanced to early-stage clinical trials. New classes of drugs include antibodies to different epitopes and forms of tau (monomeric, phosphorylated, aggregated), genetic treatments to lower tau species (antisense oligonucleotides [ASOs], small interfering RNA [siRNA], and adeno-associated viral [AAV] vectors), and small molecules to inhibit or reverse aggregation (O-GlcNAc inhibitors and direct aggregate binders). These powerful novel treatment mechanisms appear promising for testing in the AD population, as demonstrated by success in other disease populations and in preclinical models. For example, ASOs for spinal muscular atrophy have demonstrated the ability to change the course of the disease [77–79], and anti-tau therapies have demonstrated a reversal of neurodegeneration in tauopathy mouse models [80, 81]. Therefore, diversified testing of tau targets and mechanisms are essential to addressing target validation*.* Currently there are approximately 20 active tau therapeutic programs in human studies, primarily in early phase I-II trials (Alzforum, Aug 23, 2020), with the majority being passive or active immunotherapies targeting different amino acid sequences of the soluble or insoluble tau protein (Table 2). The diversity of targets reflects, in part, an incomplete understanding of how the tau protein contributes to neurodegeneration. In this section we discuss the current state of tau therapeutics in the context of recent clinical research and propose basic and translational research needed to help accelerate the discovery of effective therapies for the prevention of AD.

#### Reverse translation of tau and clinical symptoms of AD to models

Although mutations of the *microtubule associated protein tau (MAPT*, the gene encoding the tau protein), have been identified as a cause of familial frontotemporal lobar dementia-tau (FTLD-tau) [82], these mutations do not cause AD. Additionally, tau aggregates of AD [83] and FTLD (e.g., Pick’s disease) [84] are distinctly different in anatomical distribution and their clinical expression. In addition to AD and Pick’s disease, there are other clinical tauopathies (e.g., progressive supranuclear palsy, corticobasal degeneration, chronic traumatic encephalopathy), all which have distinct clinical symptoms and pathological forms of tau, highlighting the need for translational studies to account for differences between the tauopathies. Further, it is important to note that in AD, the spread of tau appears fundamentally linked to the presence of Aβ-plaques. Thus, translational studies focusing on the therapeutic targeting of tau, especially if involving mechanisms preventing tau spread, should include models that better approximate the environment of clinical AD by considering the interactions between Aβ and tau.

Another challenge is that soluble extracellular (CSF/plasma) tau increases years before insoluble/aggregated tau is detected by tau PET, and it is important to understand whether there are similar patterns in animal models. If there are similar findings in preclinical models, these would offer an opportunity to explore the potential toxicity of soluble tau [85, 86] proteins. However, there is minimal information available from animal models of AD tauopathy regarding how these CSF and plasma measures of p-tau and t-tau change [87–89]. This pattern of early soluble p-tau elevations many years prior to both cognitive decline and the development of aggregated tau indicates that (1) further mechanistic studies linking Aβ with the cellular response to tau generation are an important point for possible therapeutic focus and (2) understanding the critical links between soluble and insoluble tau pathology could have important therapeutic indications, especially for prevention. Likewise, in human studies, although the number of available tests for measuring soluble and aggregated tau has increased dramatically over the past 5 years [21, 23, 59, 61, 90], there remains a significant gap in the understanding of how soluble measures of tau are related to NFT as measured by PET. Because most studies in humans are currently cross-sectional or represent end-stage AD (e.g. post-mortem studies) there is a limited understanding of how changes in soluble tau predict or are related to the evolution of aggregated tau [91]. However, the DIAN study indicates that p-tau increases approximately 20 years before tau PET increases [59]. Animal models of tau, with a much shorter time scale and more precise understanding of the development of aggregated tau, could help to overcome some of the limits by linking measures of soluble tau to immunohistochemical measures of aggregated tau along the period of development of tau tangle pathology. For example, models have demonstrated increased tau with amyloid plaques and no tau pathology [87]. This type of work in animal or cellular models would help in planning tau therapeutic trials based on specific measures of soluble tau being targeted and how those targets relate to preventing NFT pathology.

It is important that therapeutic translational model studies should attempt to simulate the specific forms of tauopathies as closely as possible. As an example, an immunotherapy that is developed to recognize specific sites of phosphorylation or conformation of aggregated tau may have variable translatability to symptomatic AD depending on whether a transgenic *MAPT* (e.g., P301L mutation resulting in an increased propensity for aggregation of 4-repeat (4R) tau or the R406W mutation which recapitulates AD tau pathology) mutation model is used or whether an injection model of tau aggregates isolated from AD brain homogenates is used. Similarly, a therapy developed to decrease the aggregation of tau or potentially block the trans-synaptic spread and template-based ‘seeding’ of tau may have important differences based on the proportion of 3-repeat (3R) to 4-repeat (4R) tau of the host animal or cellular model and the subsequent translation to the type of tauopathy being tested in humans. Recent post-mortem work has linked the seeding activity of tau to differences in the pattern of phosphorylation and the rate of clinical progression [92] of patients. This suggests that work in cellular and animal models using tau isolated from AD patients should also take into account the heterogeneity related to the source of tissue as this could potentially affect the outcomes of studies based on the prevention of tau spread or seeding.

#### Narrowing the gap-translating biomarkers between clinical and translational models

Developments in clinical diagnostics of tau over the past 20 years have now enabled a much more favorable environment for the bidirectional translation of preclinical and clinical studies. Although the detection of soluble tau from the CSF [93–95] of patients with AD has been available for over 20 years, the majority of information on the links between tau pathology and AD has been based on post-mortem studies [55, 56, 96]. Because of the links between NFT pathology and the clinical/cognitive impairment of AD, most previous trials targeting tau have (1) been in symptomatic populations, and (2) have had clinical outcomes as the measure of efficacy (Table 2). In this scenario, the success of a tau therapy relies on the ability of a single-drug, single-target approach to have a substantial treatment effect on advanced stages of AD pathology, when tau aggregation is accelerating. Yet in most instances, preclinical studies of tau are based on more precise measurements at the tissue level (e.g. elimination, modifications, cell-cell interaction, kinase regulation) that are not translatable to human studies except, in some instances, at post-mortem [92, 97]. However, even when a similar method can be applied to both preclinical and post-mortem AD samples (e.g., immunohistochemical methods, single-cell RNA expression, tau PET), there often are important differences in the stage of disease/tau-pathological evolution when these methods are applied in the two different scenarios, potentially limiting the translatability.

One of the advantages of some animal models that develop tauopathy such those that express the P301S or P301L mutations in tau is that the mice develop not only aggregated tau pathology but also neurodegeneration with brain atrophy, synaptic and neuronal loss, and a strong neuroinflammatory responses in the tauopathy brain regions. This is similar to what is seen in humans with primary tauopathies and in AD. Thus, an effective tau related therapy would be expected to reduce progressive brain atrophy which is readily detectable by MRI in humans. However, there remain significant limitations in identifying the mechanisms that lead to the distinct tauopathy of AD (in humans)-i.e., the facilitation of tau aggregation and spread by Aβ plaques (particularly neuritic plaques), as well as the mechanisms linking the excretion of hyperphosphorylated, soluble tau to Aβ plaques. Moreover, there remain limitations in understanding the direct mechanism of toxicity of NFTs in humans, highlighting the need for studies in non-human models that can more easily be translated to human studies. One recent study demonstrates that, in a mouse model of amyloidosis, TREM2 expressed by microglia is able to limit amyloid-induced AD tau seeding and spreading as one potential mechanism tying together amyloid to tau [98]. As there are measures of soluble CSF tau in humans, preclinical models could also include similar methods of measuring TREM2 related disease progression and response to therapies. Additionally, the ultrastructural differences between AD NFTs and those of other tauopathies and how these contribute to disease onset and progression [92] all are critical considerations more amenable to preclinical mechanistic studies and could facilitate the development of more efficient prevention trials targeting tau. Table 3 outlines areas of high priority for translation of preclinical work in tauopathies to next generation prevention trials in AD.

**Table 3:** Opportunities and Challenges of translating preclinical studies of tau

Area of focus	Model	Need	Opportunity for translation
Conformational specificity of aggregated tau (disease specific)	– Transgenic models/knock in; – iPSC/iNeuron (mutation related tau/AD; non-genetic disease specific (PSP/CBD)) – Brain organoids	– Greater fidelity to AD: amyloid and tau co-pathology – Specific ultrastructural conformation of AD tau – Better evidence of soluble tau/p-tau changes (CSF/blood)	– Expand transgenic tau (MAPT) models to include mutations with evidence of AD type pathology (R406W) – Establish standards within the preclinical field of testing tau therapeutics that represent multiple conformational species of tau (≧2).
Soluble (extracellular) vs aggregated tau (biomarker validation)	– AD transgenic models (with/without tau injection paradigm) – iPSC/iNeuron	– Better understanding of the role of extracellular tau and the various truncated p-tau species (impact on neuronal function; response to stressors (e.g. extracellular amyloid) – Impact of targeting specific soluble tau species in AD prevention	– Determine extent of soluble CSF/plasma tau and p-tau profiles identified in humans with AD and tau transgenics – Preclinical studies targeting specific kinases related to amino acid specific phosphorylation – Preclinical studies targeting specific soluble p-tau isoforms
Abeta-tau interaction (AD tauopathy)	– Tau injections (AD specific) in AD transgenic models – Brain organoid – iPSC/iNeuron models (AD mutations)	– AD specific models that include both Amyloid and tau pathologies (preferably on different APOE backgrounds)	– Measurement of soluble tau in Amyloid targeted therapies
Seeding propensity of tau (tau strains)	– Transgenic models/knock in; – iPSC/iNeuron (mutation related tau/AD; non-genetic disease specific (PSP/CBD/sporadic AD)) – Brain organoids (mutation related tau/AD; non-genetic disease specific (PSP/CBD/sporadic AD))		– Use of Tau PET in preclinical studies (same tracers as in clinical studies) – Standardize methods for determining seeding propensity of tau for consistent reference across the field – Include multiple AD patient tau “seeds” in preclinical models that are representative of various clinical features (rapidly progressive vs slowly progressive)

### Summary

The association between misfolded tau and the clinical symptoms of AD prioritizes tau for developing treatments in AD. Yet, the recent history of Aβ specific therapies illustrates the immense challenge of targeting misfolded proteins in neurodegeneration. Recent advancements in tau biomarkers, in pre-symptomatic and symptomatic AD has provided the opportunity for a more precise approach in preclinical models. Essential to this is the use of models that better reflect the tauopathy of AD and consideration of how to also adapt mechanistic studies that integrate tools and biomarkers currently available in clinical studies.

Finally, the discovery and validation of AD blood-based biomarkers, made possible with the development of novel techniques (e.g. immunoprecipitation–mass spectrometry or the high-precision immunoassays) [23, 25, 99–101] will offer major opportunities for screening and enrollment of potential participants for primary and secondary prevention trials, reducing trial duration and costs due to screen failures.

## Additional targets

### Therapeutic approaches targeting inflammation and microglia

Neuroinflammation is now recognized as a prominent feature in the neurodegeneration process leading to symptomatic AD [102–106]. Microglial and astrocytic activation are thought to play a major role in the initiation and exacerbation of CNS inflammation in response to AD pathology [107, 108]. Although classical neuropathological lesions in AD include neuritic plaques composed of Aβ and intraneuronal accumulation of NFTs featuring hyperphosphorylated tau [54, 109], numerous studies describe the presence of activated glial cells in the vicinity of the plaques. The spatial relation between Aβ and reactive glial cells suggests that glial cells are activated by aggregated Aβ [110], triggering an inflammatory response at the early stages of the disease (before symptomatic phases). Several AD models consider that early glial activation may be protective by facilitating Aβ phagocytosis and degradation. However, the chronic inflammatory response related to glial activation may be deleterious, leading to acute neuronal membrane damage, neurotoxicity, and neurodegeneration at later stages of the disease. Moreover, microglia activation reduces the formation of neuritic dystrophy surrounding amyloid [111–113], creating a continuous cycle of molecular and cellular events that influences AD pathology progression and a neuroinflammatory response throughout the disease process. In a similar fashion, gliosis has been reported in animal models and other tauopathies in the absence of Aβ pathology, suggesting a tau-dependent microglial activation that maintains the glial inflammatory response through the disease [114]. In summary, it is likely that Aβ and tau synergistically contribute to neuroinflammation and neurodegeneration via chronic glial activation, but the inflammatory response has different roles at different stages of the disease.

The presence of early and chronic inflammation in AD via microglial activation supports the use of immunomodulating agents as potential therapies aimed at disease treatment, but the optimal timing of such intervention using such candidates is still under debate. Immunomodulating therapies might have different effects depending on the state disease; inhibition of microglial function by anti-inflammatory approaches may prove detrimental during early phases of Aβ aggregation; however, it may reduce neurodegeneration during later phases of Aβ aggregation and especially the NFT phases [114].

Different strategies are currently under development aimed at modulating immune cell function in neuroinflammation. Numerous studies have explored the use of non-steroidal anti-inflammatory drugs (NSAIDs); however, despite compelling evidence in animal models highlighting the protective effects of NSAIDs in AD, clinical trials using these compounds for AD treatment have been mostly disappointing, probably related to the inclusion of participants too late into the disease course and the differential effect of immunomodulation depending on disease stage as well as the lack of CNS target engagement [115–117]. As indicated by secondary analyses from the Alzheimer’s Disease Anti-inflammatory Prevention Trial (ADAPT), the effect of NSAIDs may differ according to disease stage; asymptomatic individuals treated with naproxen showed reduced AD incidence, whereas those at later stages of AD experienced faster cognitive decline [118, 119]. Nevertheless, it’s still controversial whether NSAIDs will provide a meaningful long-term clinical benefit [120].

Other promising candidates for inflammatory response regulation include proteins involved in microglial function and inflammation (e.g., TREM2, APOE, CD33, CR1, ABCA7 and SHIP1). The recent identification of TREM2 variants as late-onset AD (LOAD) genetic risk factors has prompted several studies to determine the role of TREM2 in glial regulation and inflammatory response. TREM2 seems to play a key role in microglial recruitment, phagocytosis, and clearance of Aβ [121, 122]. Several reports AD suggest that TREM2 deficiency results in decreased microglial activation and a subsequent reduction of plaque-associated microglia, which augments local Aβ toxicity and amyloid-induced neuritic dystrophy. While these studies support augmenting TREM function in the amyloid stage of preclinical AD, other studies show that decreasing TREM2 function in tauopathy models decreases brain atrophy and synaptic loss [123, 124]. So the timing of when to target TREM2 and the therapeutic mechanism (activation or inhibition) to treat AD pathology remains to be determined depending on disease stage [112].

Ultimately, the successful implementation of immunomodulating therapies might lie in maintaining the fine balance between reparative and damaging functions but will almost certainly be a component of combination therapies for AD.

### Therapeutic approaches targeting apolipoprotein E function

*APOE4* is the most prevalent genetic risk factor for sAD, with several studies pointing out a strong relationship between *APOE*-ε4 and AD pathology [125, 126]. *APOE4* carriers are more likely to develop AD several years earlier relative to *APOE3* and with a dose-dependent effect. Conversely, *APOE2* carriers have a ‘protective’ effect relative to *APOE3* and *APOE4* carriers, most likely through both Aβ-dependent and other independent mechanisms yet to be determined [127]. These findings suggest a possible toxic effect of APOE-ε4 in the brain, providing an avenue to delay or stop the development of AD via blocking APOE-ε4 expression. Current concepts for APOE-targeted AD therapies include: (1) regulation of ApoE levels; (2) modification of ApoE properties or structure; (3) re-programing APOE function via gene editing and (4) indirect therapeutic approaches via ApoE receptor modification, maintaining vasculature integrity, and inflammatory systems. More detailed information regarding APOE-targeted AD therapies are provided in previous reviews [128–130].

However, there are some limitations to these approaches, including: (1) The percentage of people with at least one APOE-ε4 allele in the population is ~25% and the relation between APOE4 and dementia risk varies according to population admixture [127, 131, 132], therefore the number of individuals to benefit from this approach may be somewhat limited in a prevention platform. It should be noted, however, that ~65% of people with symptomatic AD carry at least one E4 allele; (2) although APOE isoforms have been implicated in numerous processes, including crosstalk with Aβ [133, 134], tau phosphorylation [126], lipid metabolism, vascular function [135, 136] and inflammation [137], the molecular mechanisms that mediate the pathological effects of APOE-ε4 in AD development remain to be determined; (3) most animals models in AD express non-physiological levels of expression of Aβ and tau, thus it is difficult to assess if findings from *APOE* modification in these models will yield similar results in human clinical trials. Answers to these questions via better understanding of APOE modification in animal models may pave the way for a more targeted approach to APOE-based therapies in AD.

Despite our increased understanding of the detrimental effect of APOE-ε4, there is also sufficient evidence of a protective effect of APOE-ε2 against AD [127, 138]; therefore, it is reasonable to consider that APOE-ε2 based therapies may yield significant therapeutic effects in APOE-ε4 and APOE-ε3 individuals. Viral-mediated overexpression of APOE2 or converting APOE-ε4 to APOE-ε2 via gene-editing tools such as the CRISPR-Cas system may constitute cost-effective targets for primary and secondary prevention trials in APOE-ε4 and APOE-ε3 individuals. However, the mechanism underlying the protective effect of APOE-ε2 has remain mostly elusive and more evidence from animal models will be required before fully proceeding to prevention trials. Due to the duration of these trials, a better understanding of the long term effects of APOE-ε2 overexpression is required, including for possible detrimental effects such as increased risk for stroke [139–141], CAA [142, 143] and age related macular degeneration [144, 145]. Supporting for interventions that aim to modify or edit APOE comes from the recent report that homozygosis for the APOE3 Christchurch variant (R136S) markedly delayed cognitive decline in a single presenilin 1 (PSEN1) mutation carrier [146]. However, these findings will warrant further replication in animal models before moving to therapeutic trials. Furthermore, whether the protective effect of the APOE3 Christchurch variant is through disruption of tau spread or decreasing the ability of APOE to initiate a microglial-mediated inflammatory response to tau pathology remains to be determined.

## Combination therapies in prevention trials

The conceptualization of AD as a chronic illness of over 20 years duration supports these two hypotheses: (1) mechanism-based therapies for AD will have optimal benefit when initiated in the asymptomatic stage, prior to substantial damage to synapses and neurons (see previous sections); and (2) once the AD pathological cascade has been initiated, a combination of therapies that together target multiple aspects of AD pathology will be more effective than monotherapies that address only a single abnormal factor (e.g., the cerebral accumulation of aggregated Aβ). Although the complex biological mechanisms leading to AD (e.g., Aβ plaques, NFTs, and inflammation) suggest that the disease may be more effectively treated with a combination approach rather than a single therapy, to date most AD trials have evaluated a putative disease-modifying monotherapy (without success). Thus, there is a need for new and more innovative study designs, including the implementation of combination trials. The issues pertinent to combination therapy in AD are comparable to combination therapies that are used in other chronic illnesses such as cancer and cardiovascular disease.

A Cochrane review of five trials in individuals with symptomatic AD with a range of Mini Mental State Examination [147] scores from 5 to 22 found a small but significant benefit of the combination of an approved cholinesterase inhibitor and memantine for global, cognitive, and behavioral measures but no benefit for instrumental and basic activities of daily living [148]. Hence, combination therapy with “standard of care” medications provides at best an uncertain clinical benefit [148]. Greater efficacy may be realized with combinations of mechanism-based therapies. Combination therapies can be evaluated with an add-on trial design, where the effects of a new therapy are compared with placebo on the background of a known effective therapy for AD. Because no anti-AD therapy has yet demonstrated efficacy, the focus here will be on combination therapies that feature a 2 × 2 factorial trial design, in which each of 2 drugs (addressing different targets) are tested alone and in combination versus placebo [149].

Aβ remains an important target [150] for disease-modifying therapies in AD, although to date, clinical trials of anti-Aβ monotherapies in persons with symptomatic AD have failed to clearly demonstrate clinical efficacy. However, on the basis of removing amyloid plaques, the US FDA recently gave accelerated approval of aducanumab,  thus further enabling combination treatments to include a treatment to remove amyloid plaques. A combination therapy for AD might address different points in the pathway leading to deposits of aggregated Aβ-42, such as a monoclonal antibody directed toward Aβ-42 and a β-secretase (β-site APP cleaving enzyme 1, or BACE) inhibitor to both remove deposited amyloid via the antibody and reduce the generation of new amyloidogenic isoforms with the BACE inhibitor. Unexpected cognitive worsening, as well as other potential adverse effects (e.g., hepatotoxicity, weight loss or neuropsychiatric symptoms), with BACE inhibitors in phase 2/3 trials have halted their therapeutic development in AD [11, 12, 151], but the model targeting the amyloid pathway at different stages remains a viable strategy, and may be facilitated by next generation gamma secretase modulators [152, 153]. Another approach to combination therapy is to target two (or more) pathogenic pathways. Anti-tau agents already are in clinical trial, leading to consideration of combining anti-amyloid and anti-tau therapies [154]. Other potential combinations of therapies that have diverse mechanisms of action could include agents that address neuroinflammation, apolipoprotein E, mitochondrial modifiers, free radicals, autophagy, or the disrupted blood-brain barrier [44, 155]. In addition, future studies should explore the potential role of human induced pluripotent stem cells (hiPSCs) in AD treatment, however to improve the interpretation of hiPSC experiments, more effective animal models and access to primary or age-matched cells from the human CNS are needed (Table 3).

Current trials should take into consideration the significant evidence of multiple pathologies co-occurring with AD, including vascular brain injury, cerebral amyloid angiopathy (CAA), Lewy body pathology and TDP-43 inclusions [156–158]. Presence of AD co-pathologies may influence neurodegeneration, lowering the threshold and contribute to faster cognitive decline; which further supports the relevance of combinations therapies aimed to slow cognitive decline. Therapeutic interventions should also be coupled with lifestyle interventions to reduce dementia risk. As shown by the Finnish Geriatric Intervention Study to Prevent Cognitive Impairment and Disability (FINGER) study, multidomain lifestyle intervention (e.g., physical exercise, a healthy diet, cognitive stimulation) could improve or maintain cognitive functioning in at-risk elderly individuals [159]. Similar to the FINGER study, the US study to Protect Brain Health Through Lifestyle Intervention to Reduce Risk (US-POINTER), will test whether a similar 2-year intensive lifestyle intervention, adapted to American culture, can protect cognitive function in older adults in the U.S. [160] Several global initiatives are under way, under the World-Wide FINGERS Network aimed to determine whether lifestyle interventions that simultaneously target many risk factors protect cognitive function in older adults who are at increased risk for cognitive decline [160, 161]. Related to studies of lifestyle interventions, similar rigor of non-human, mechanistic studies should also be applied to better understand the mechanisms of potential clinical benefits of exercise, diet and cognitive stimulation. Finally, as we advanced in AD therapeutic strategies, clinical trials should enroll and follow participants from all ethnic and racial groups so that the results are applicable to all.

Determining which mechanisms to target will depend on multiple factors, including the availability of appropriate drugs with preclinical evidence of efficacious target engagement and an acceptable safety profile, the stage of AD (preclinical or symptomatic), and potentially a precision medicine approach to identify individual mechanistic pathways that then would determine a specific combination of drugs to target relevant pathways [44].

The use of combination therapies has already proven to be successful in complex diseases like rheumatoid arthritis, cancer, tuberculosis, and HIV/AIDS, and are also likely to increase the odds for success in AD therapy development. However, the roadmap for combination therapies in AD will require addressing several challenges including: (1) lack of predictable animal models to test multiple targets (e.g., newer AD animal models will be required to express multiple disease mechanisms), (2) determination of which pathways to target based on disease stage (e.g., ideally, each drug in a combination therapy must target distinct disease pathways and be stage-specific, such as having one drug target Aβ plaque removal and another prevent tau tangle formation in early stages of the disease) (3) how to handle complex clinical trial designs to determine additive versus synergistic treatment effects of two or more novel therapies, and (4) addressing safety concerns about additive toxicity from drugs administered simultaneously. Finally, although there are many as yet unresolved issues to address, including timing of the intervention, duration of therapy, and adverse events of the therapeutic agents, trials of combination therapies should be developed not only to evaluate efficacy in persons with symptomatic AD but also for secondary prevention in cognitively normal individuals with biomarker evidence of preclinical AD. It is possible to use molecular biomarkers to “stage” preclinical AD and identify individuals who are most at risk of developing symptomatic AD within a defined observation period [162]; new biomarkers may further inform that risk [68].

Prevention trials of combination therapies may offer greater chances of benefit, in either delaying or even preventing the onset of symptomatic AD, than trying to reverse or halt cognitive and functional decline in individuals who already have symptomatic AD and thus have experienced irreversible cerebral damage.

## Conclusions

AD prevention trials have the potential and promise to achieve highly successful therapeutic goals of delaying or preventing AD dementia. The impact of preserving cognitive independence is enormous and justifies the currently challenging and uncertain initial prevention efforts. Basic and Translational recommendations to advance AD prevention are shown in Table 4. AD prevention challenges that must be met are defining optimal targets, when and what asymptomatic stage to target the different pathologies, developing relevant AD prevention model studies that inform clinical prevention strategies, and the informed design of combination interventions for this complex neurodegenerative disorder. These are all attainable aims that require the investment of basic, translational and clinical researchers working together with the common goal of maximally informing clinical prevention efforts. Although developing prevention approaches can be challenging, the substantial long-term benefits of cholesterol-lowering statins in individuals at high risk of cardiovascular disease provides an instructive lesson about prevention which can be replicated in AD. The approach is being taken in AD by defining targets based on necessary and sufficient conditions to cause disease (increased cholesterol/atherosclerosis vs. amyloid/tau aggregation), and then treating with interventions that have large impacts on the target, at a stage of disease before downstream or end-organ damage occurs. As a field, if we are successful in this prevention approach, years to decades of independent living may be provided to millions of at-risk people.

**Table 4:** Basic and Translational recommendations to advance AD prevention trials

**1) Target validation**	Demonstrate necessary or sufficient factors for developing AD pathology and disease (e.g. ApoE, TREM2, mutations in PSEN1, PSEN2, APP) Discover human variations that negate risk for AD (e.g. Icelandic and ApoE mutations) Determine the atomic models of AD pathology for amyloid plaques and other associated pathologies (e.g. synapse and neuron loss, alpha-synuclein, TDP-43) as has been accomplished for tau tangles. Identify the relationships between biological changes and consequences of amyloid, tau, and neurodegeneration changes as it relates to AD and clinical manifestation. Diversified testing of tau targets and mechanisms are essential to addressing target validation.
**2) Animal and** ***in vitro*** **model development**	Simulate and model the different forms of tauopathies (AD vs. 4R tauopathies) to emulate the molecular and structural pathology present in each disease. Both *in vitro* and animal *in vivo* preclinical model studies need to match the disease to inform clinical trial design. Simulate and model Aβ amyloid plaque and other isoform changes with human AD stages. Implement standardization protocols for testing Aβ therapies by stage of disease to include primary prevention (pre-plaque), secondary prevention (plaque growth stage before tangles), symptomatic (fully established amyloid plaque load with downstream consequences in tau aggregation and neurodegeneration) in transgenic or other related *in vivo* models. Create greater fidelity of AD in animal models: amyloid and tau co-pathology; specific ultrastructural conformation of AD tau and better recapitulate the sequence of stages, for example, soluble tau and p-tau changes in CSF and blood. Develop models of AD inflammation and microglial activity that mimics the specific AD related inflammation and neurodegeneration. Develop standard assays and techniques to measure drug effects on pathology, pharmacodynamics, and pharmacokinetics in animal and cellular models that are most directly translatable to human clinical studies. Accelerate studies and programs of preclinical models that can test rational combinations with a focus on translating these to prevention trials. For example, removing amyloid while preventing the spread of tau pathology.
**3) Biomarker development**	Develop novel biomarkers that can track pre-clinical biological changes and distinguish stages of pre-clinical AD and predict future biological and clinical changes. Understand the relationship between currently available biomarkers and the pathophysiology of the AD process and how this relates to pathology and current and future clinical measures. For example, how do different phosphorylated tau species related to tau aggregation in the brain and to clinical onset and progression? Improve understanding of biomarkers relation to clinical symptoms and age at onset. For example, track the longitudinal changes in amyloid-beta, soluble tau species vs. aggregated tau changes
**4) Prevention trial design**	Improve prevention trial screening accuracy through the development and implementation of cost-effective non-invasive biomarkers. What combination of biomarkers are optimal for identifying stage of disease, years to clinical onset and decline and prognostic of rates of decline? Identify stages of asymptomatic disease that match the pathophysiology with the intervention target. For example, intervening in tau spread during rapid tau aggregation growth, or blocking the amyloid-tau link before tau pathology becomes autonomous. Develop sensitive cognitive measures aimed at demonstrating efficacy in the very earliest detectable stages of AD. Novel approaches may need to include rapid and frequent sampling and significantly complex cognitive tasks to accurately track asymptomatic cognitive dysfunction.

## Data Availability

Not applicable.

## Abbreviations

AAV: Adeno-associated virus
AD: Alzheimer’s disease
ALS: Amyotrophic lateral sclerosis
ApoE: Apolipoprotein E
APP: Amyloid precursor protein
ASOs: Antisense oligonucleotides
Aβ: Amyloid-β peptide
BACE: β-Site amyloid precursor protein cleaving enzyme
CNS: Central nervous system
CRISPR: Clustered regularly interspaced short palindromic repeats
CSF: Cerebral spinal fluid
DIAN-TU: Dominant Inherited Alzheimer Network Trials Units
FTLD: Frontotemporal lobar dementia
GSI: γ-Secretase inhibitors
hiPSCs: Human induced pluripotent stem cells
LOAD: Late-onset Alzheimer disease
MAPT: Microtubule associated protein tau
NFTs: Neurofibrillary tangles
NSAIDs: Non-steroidal anti-inflammatory drugs
PSEN: Presenilin
sAD: Sporadic Alzheimer disease
siRNA: Small interfering RNA
TREM: Triggering receptors expressed on myeloid cells
